# Antipsychotic Medication-Induced Hyperthermia Leading to Cerebrovascular Accident: A Case Report

**DOI:** 10.7759/cureus.18651

**Published:** 2021-10-11

**Authors:** Karimah Best, Dena H Tran, Camille Bulte, Avelino C Verceles, Miriam B Michael

**Affiliations:** 1 Internal Medicine, American University of Antigua, Osbourn, ATG; 2 Internal Medicine, University of Maryland Medical Center Midtown Campus, Baltimore, USA; 3 Pulmonary and Critical Care Medicine, University of Maryland School of Medicine, Baltimore, USA; 4 Internal Medicine, Howard University, Washington, DC, USA; 5 Internal Medicine, University of Maryland, Baltimore, USA

**Keywords:** heat stroke, schizophrenia and other psychotic disorders, cerebrovascular accident, hyperthermia, antipsychotic

## Abstract

Antipsychotic medications are used in the management of schizophrenia. Antipsychotic medications treat both positive and negative symptoms via the dopamine D2 receptor and serotonin 5-HT2A blockade pathway. Side effects include hyperprolactinemia, prolonged QTc, and neuroleptic malignant syndrome. However, antipsychotic medication-induced hyperthermia potentiating a cerebrovascular accident (CVA) is a rare side effect that is less well known.

A 47-year-old male presented to the emergency department (ED) via emergency medical services for altered mental status. He was given naloxone without improvement in mental status. His glucose was 110 mg/dL. Upon presentation to the ED, he was hyperthermic (106.7 degrees Fahrenheit) and tachycardic (heart rate of 160’s beats/minute). Home medications included risperidone and fluphenazine. After the resolution of his hyperthermia, he had a right-sided facial droop concerning a cerebrovascular accident. Magnetic resonance imaging (MRI) of the brain confirmed an early/acute subacute right cerebellar infarction. The patient received optimal treatment; his mental status returned to baseline, and he was discharged home without antipsychotic medications.

Patients who are prescribed antipsychotics should be aware of the potentially fatal adverse events that can occur from these medications. Thermoregulation may be impaired in these patients, resulting in significant hyperthermia, in which case antipsychotic medications should be discontinued.

## Introduction

Antipsychotic medications treat both positive and negative symptoms due to their potent dopamine D2 receptor blockade and serotonin 5-HT2A inhibition [1]. Side effects, including hyperprolactinemia, prolonged QTc, and neuroleptic malignant syndrome, have been well described in the literature. Antipsychotic medications are also well known to increase the risk for developing hyperthermia, as evidenced by multiple reported cases of antipsychotic-induced heatstroke [2,3]. 

In addition to cardiac dysfunction, hypotension, rhabdomyolysis, renal and hepatic injury, and seizures, heatstroke can lead to cerebral edema and cerebrovascular injury, including acute ischemic infarcts. These psychotropic drugs likely impair an individual’s ability to thermoregulate, resulting in heatstroke and an insinuating cerebellar syndrome. Limb and gait ataxia, dysarthria, dysphagia, hemiplegia, and oculomotor disturbances are often manifestations of a cerebellar infarction [4]. We present a case of a 47-year-old male who presented with hyperthermia resulting in a cerebrovascular accident (CVA) due to the use of antipsychotic medications for his psychiatric history. The patient received optimal treatment; his mental status returned to baseline, and he was discharged home without antipsychotic medications.

## Case presentation

We present a case of a 47-year-old male with a medical history significant for schizophrenia and intellectual disability who presented to the emergency department (ED) for altered mental status. He was a resident at an adult day care program. Home medications include risperidone 2 mg in the morning and 4 mg at night, fluphenazine 5 mg daily, benztropine 1 mg twice daily, and valproic acid 250 mg twice daily. As per his caretakers at the adult day care program, the patient is usually awake; alert; oriented to person, place, and time; and interactive, with no focal neurological deficits. Emergency medical services administered intranasal naloxone 6 mg with minimal effect. The patient remained minimally responsive on the initial presentation with a Glasgow Coma Scale score of 9. There was initial concern for heatstroke as the patient was found down on the streets; however, the outdoor temperature was in the 90’s degrees Fahrenheit. Physical examination was notable for hyperthermia with a rectal temperature of 106.7 degrees Fahrenheit, blood pressure of 110/68 mmHg, heart rate of 160’s beats/minute, respiratory rate of 27 breaths/minute, and oxygen saturation of 90% on room air. The patient was grimacing to voices and spontaneously moving all four limbs but was otherwise unable to follow commands. Pupils were 3 mm equal, round, and reactive to light. There was no muscle rigidity. The laboratory results were remarkable for white blood cell count of 13 K/mcL; potassium level of 3.1 mmol/L, suggesting hypokalemia; phosphate level of 0.6 mmol/L, suggesting hypophosphatemia; blood urea nitrogen of 9 mg/dL; creatinine of 1.48 mg/dL; corrected calcium level of 7.22 mg/dL, suggesting hypocalcemia; albumin of 2.6 g/dL; troponin of 0.278 ng/mL; elevated creatinine kinase of 7,827 U/L; and lactate of 1.4 mmol/L. Electrocardiography (EKG) showed sinus tachycardia with no ST-segment changes, suggestive of active ischemia. Urine toxicology was negative. Computed tomography (CT) of the head was negative for acute intracranial pathology, and CT of the chest showed mild interstitial prominence. 

In the ED, the patient received 2 L intravenous fluids and 1 g calcium gluconate intravenously and underwent active cooling with ice packs and cool water bladder irrigation. His hyperthermia improved to 102.6 degrees Fahrenheit within one hour, and his heart rate improved to 110’s beats/minute. The patient became more responsive to noxious stimuli. Shortly thereafter, the patient had a disconjugate gaze with extensor posturing and generalized tonic-clonic activity. He was given lorazepam and loaded with levetiracetam, which broke his seizure episode.

The patient was stabilized and admitted to the intensive care unit for further management. EKG showed sinus tachycardia. The patient continued to be somnolent but was more arousable since admission and was awake and alert, and oriented to person and place. He was afebrile at the time. Physical examination was negative for any focal neurological deficits. Creatinine kinase improved after intravenous hydration. 

**Figure 1:**
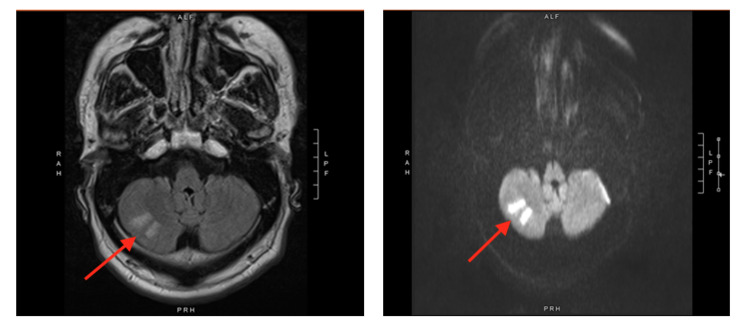
Magnetic Resonance Imaging of the Brain Red arrows depict acute/early subacute infarcts in the inferior aspect of the right cerebellum hemisphere.

On hospital day 4, the patient had slurred speech, right-sided facial droop, right-sided tongue deviation, and right-sided upper and lower extremity weakness, raising suspicion for a cerebrovascular accident (CVA). He did not have nystagmus, dysmetria, or coordination and gait abnormalities. The patient did not endorse dysphagia or drooling, and his uvula was midline. His strength was 4/5 on the right upper and lower extremities compared with 5/5 on the left upper and lower extremities. His handgrip strength was also decreased on the right side. Magnetic resonance imaging (MRI) of the brain showed acute/early subacute infarcts in the inferior aspect of the right cerebellum hemisphere without hemorrhage or mass effect (Figure 1). Echocardiogram was negative for a patent foramen ovale, and carotid duplex was normal. Neurology was consulted who did not recommend clopidogrel or thrombolytics given unknown last well-known time. The patient was medically managed with aspirin 81 mg and atorvastatin 40 mg. The patient’s mental status returned to baseline, and he was discharged without antipsychotic medications.

## Discussion

**Figure 2:**
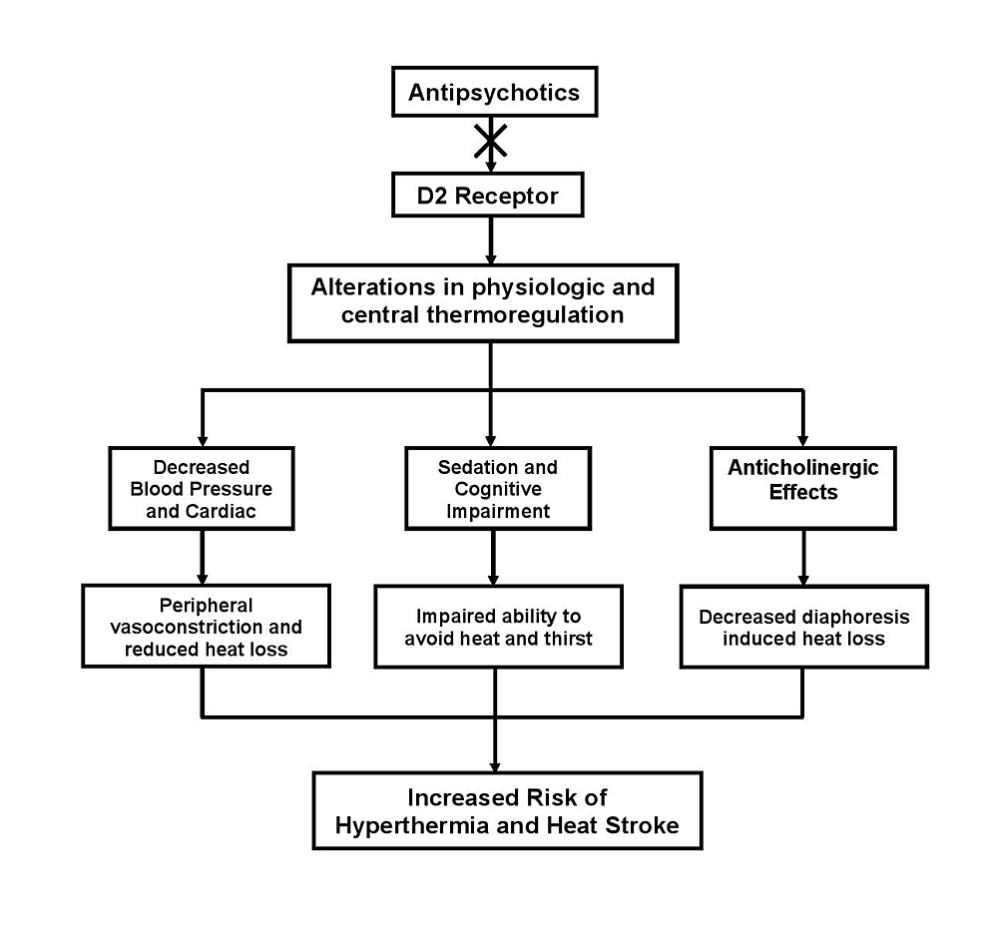
Proposed Mechanism of Action of Antipsychotic Medications Antipsychotic medications alter physiologic and central thermoregulation and subsequent proposed pathways.

We present a case of significant hyperthermia in a psychiatric patient who was prescribed first- and second-generation antipsychotics resulting in a cerebrovascular accident. We speculate that the cerebellar damage was a direct result of the hyperthermic state incited by his antipsychotic medications risperidone and fluphenazine. Hyperthermia is a rare but well-known side effect of risperidone and results in an increased risk of developing heatstroke [3]. The mechanism of action is unknown but may be due to the alteration of neurotransmitters such as dopamine and serotonin, leading to abnormal physiologic and behavioral responses to central thermoregulation, general sedation, and cognitive impairment (Figure 2). This may impede one’s ability to perceive and therefore avoid excessive heat and thirst, leading to hypotension and reduced cardiac output. The compensatory peripheral vasoconstriction and reduced heat loss result in decreased diaphoresis and inefficient cooling and thermoregulation due to anticholinergic effects [2].

Patients with schizophrenia are often managed with potent antipsychotics that inhibit D2 receptors that alter the neurovascular units in the frontal cortex [5]. These psychotropic medications likely impair an individual’s ability to thermoregulate, resulting in heatstroke and the insinuating cerebellar syndrome [4]. Additionally, hyperthermia in a CVA is a result of a simultaneous blockade in the hypothalamus and basal ganglia, impairing heat dissipation and facilitating thermogenesis and rigidity [4].

There have been case reports of hospitalization due to hyperthermia from psychotropic drugs causing heat-related mortality [2]. The thermal injury starts at temperatures of 107.6 degrees Fahrenheit; however, neurological symptoms and organ dysfunction occur above 105.8 degrees Fahrenheit, and the cerebellum and Purkinje cells are most susceptible to hyperthermia [3,4]. Hsu et al. performed a meta-analysis and showed that 17,00 out of 186,188 antipsychotic medication users had cerebrovascular accidents [6]. As observed in our patient, his temperature peaked at 106.7 degrees Fahrenheit, making him susceptible to neurological damage. 

## Conclusions

Patients who are prescribed antipsychotics should be aware of the potential adverse events that could be fatal. Thermoregulation may be impaired in these patients, resulting in significant hyperthermia, and the antipsychotic medications should be discontinued.
